# Antipredator behavior of European rabbits by day and night

**DOI:** 10.1093/beheco/arag123

**Published:** 2026-10-16

**Authors:** Sam A Di Stefano, Michael A Weston, Roan D Plotz, Joel Krauss, Kaori Yokochi, Anthony R Rendall

**Affiliations:** School of Life and Environmental Sciences, Faculty of Science, Engineering and the Built Environment, Deakin University, 221 Burwood Hwy, Burwood, Victoria 3124, Australia; School of Life and Environmental Sciences, Faculty of Science, Engineering and the Built Environment, Deakin University, 221 Burwood Hwy, Burwood, Victoria 3124, Australia; Deakin Centre for Marine Science, School of Life and Environmental Sciences, Deakin University, 75 Pigdons Road, Waurn Ponds, Victoria 3220, Australia; School of Life and Environmental Sciences, Faculty of Science, Engineering and the Built Environment, Deakin University, 221 Burwood Hwy, Burwood, Victoria 3124, Australia; School of Life and Environmental Sciences, Faculty of Science, Engineering and the Built Environment, Deakin University, 221 Burwood Hwy, Burwood, Victoria 3124, Australia; School of Life and Environmental Sciences, Faculty of Science, Engineering and the Built Environment, Deakin University, 221 Burwood Hwy, Burwood, Victoria 3124, Australia; School of Life and Environmental Sciences, Faculty of Science, Engineering and the Built Environment, Deakin University, 221 Burwood Hwy, Burwood, Victoria 3124, Australia

**Keywords:** fear, escape ecology, lagomorphs, nocturnality, rabbit

## Abstract

Most fear ecology research focusses on daytime escape, yet nighttime escape is also likely to be critical to survival. Current research on fear ecology is predicated on the assumption that escape is the same by day and night, yet recent research alludes to greater complexity in responses. Many prey species are active at night to avoid predators, some predators are nocturnal, and prey likely must rely on a different mix of sensory modalities to detect approaching predators at night. By incorporating thermal imaging technology, we measured alertness, distance to cover and Flight-initiation Distances (FIDs) by day and night, across different predator regimes of European Rabbit *Oryctolagus cuniculus*, an invasive species in Australia with a long history of coexistence with predators. Distance to cover increased by night, decreased with rabbit alertness, and increased with Starting Distance (SD). The probability of being alert increased closer to cover during day and night. FIDs were longer with longer SDs. Rabbits fled more directly towards cover the further they were from cover at night (but not day) time, and only in the absence of cats and foxes. We demonstrate intense antipredator behavior of rabbits by day and night, and specific nocturnal antipredator adaptations, such as using more open areas and only escaping towards cover where terrestrial nocturnal predators are absent. Given the nuanced changes in antipredator behavior by day and night, our results call for future studies to compare daytime and nighttime behaviors.

## Teaser

We explored the escape behavior of European rabbits when approached by a person during daytime and nighttime. Rabbits were further from cover by night than by day and were more alert when near cover. Rabbits fled more directly towards cover the further they were from cover only at night and in the absence of cats and foxes.

## Introduction

Escape from predators influences whether individuals, or even species, persist or perish and is therefore under strong evolutionary selection. A species’ capacity to escape predators is based on its evolved and/or learned antipredator behaviors (Cooper and Blumstein 2015). A host of factors influence variation in escape behavior between species, yet within species variation is also typically high (Weston et al. 2012; Cooper and Blumstein 2015). Given the demonstrated differences between conspecific populations in their propensity to escape (Stankowich 2008), combined with reports of at least some level of capacity to habituate within individuals of some species (Runyan and Blumstein 2004), understanding variation in escape within and between populations offers key insights into drivers of escape behavior. Recent advances in the understanding of escape ecology have suggested two prominent themes with respect to within-species patterns; namely escape behavior can vary in relation to: (i) space (ie between areas or “landscapes of fear”; Mendes et al. 2020), and (ii) in time (ie with season, time of day etc.; Ekanayake et al. 2022).

Where predator risk varies in time and space, prey may avoid predators. Clear and compelling evidence of avoidance of predators by prey is available for some species which can alter space use and/or temporal activity patterns to avoid or reduce encounter rates with predators (Miritis et al. 2020; Eketone et al. 2026). For example, northern giraffes (*Giraffa camelopardalis*), Burchell's zebras (*Equus burchelli*), and blue wildebeest (*Connochaetes taurinus*) in South Africa actively avoided areas where lion (*Panthera leo*) and leopard (*Panthera pardus*) space use were high (Thaker et al. 2011). Examples of temporal avoidance of predator risk also exist, especially for mammals (including putative predators such as brown bears *Ursus arctos*), and include temporal avoidance of humans which are known predators themselves (Gaynor et al. 2018; Ordiz et al. 2019). However, for many prey species, predator avoidance is impossible or incomplete due to factors such as limited mobility, social constraints (eg territories, competition, etc.), and/or specific resource needs. Such species must therefore cope with in situ predator risk, which likely varies across space and time (Moseby et al. 2023).

Temporal variation in predator risk can involve factors associated with prey such as specific escape/defence when breeding (Sanchez-Gomez et al. 2024), seasonal life history events such as migration (Mikula et al. 2018), prevailing energy states (Lagos et al. 2009), diel activity patterns (Sogliani et al. 2021) and use of refuges when inactive (van der Vinne et al. 2019). Predators often exhibit seasonality or diel activity patterns themselves; indeed, many predators are nocturnal or nocturnally active (Ikeda et al. 2016; Rees et al. 2024). In comparison, cathemeral prey species face a particular challenge in relation to predators; they must manage their risk in drastically different sensory and predator regimes, that is in light and in darkness (Clarke et al. 1995; Blumstein 2002; Steer and Doody 2009). Nighttime behavioral ecology remains understudied but could be disproportionately driving evolution and ecological form and function (Gaston 2019). Escape of prey from predators at night is poorly understood compared with daytime escape behavior, largely due to sensory impediments to researchers at night (Gaston 2019). However, technology now permits more effective nocturnal escape and fear ecology research (Rendall et al. 2024).

### Escaping predators by day and night

Nocturnal species may need to rely on different mechanisms of detecting predators when compared with diurnal species. For example, eastern gray kangaroos (*Macropus giganteus*) often congregate when foraging by night and often in closer proximity to cover, a behavior thought to assist in mitigating predation risk by the invasive red fox (*Vulpes vulpes*) (Banks 2001). In contrast, by day kangaroos often seek shelter and refuge, avoiding high temperatures, but also keeping juveniles safe from aerial predators (ie, wedge-tailed eagles *Aquila audax*; Jarman 1991). Behaviors, mechanisms of detecting threats, and modes of escape are therefore likely to change during daytime and nighttime, driven by changing predator regimes and altered perceived risk states.

Nighttime escape, much like daytime escape, varies with context. Nocturnal environments are not constant; moon phase can greatly influence ambient light levels and have been shown to influence animal behavior (Sharpe and Goldingay 2019; Linley et al. 2021; Sogliani et al. 2025) including perceived risk (Welch et al. 2017). Artificial Light at Night (ALAN) also increases in proximity to humans and can more permanently alter ambient light conditions having a diverse variety of impacts on wildlife (Finch et al. 2020); and has been found to cause a step-change in the escape behavior of Silver Gulls (*Chroicocephalus novaehollandiae*; Weston and Yokochi 2025). Traill et al. (2016) found that blue wildebeest and plains zebra (*Equus quagga*) made greater adjustments to their movements when they were in proximity to lions in relation to different lunar phases (full and new moon). Although perceived risk may vary with prevailing light levels, animals must balance this against the cooccurring threat of predation.

### The European rabbit: a model species

European rabbits (*Oryctolagus cuniculus*) are cathemeral and have highly flexible antipredator behaviors, having coevolved in their native range with a host of predators (Barrio et al. 2010; Atkins et al. 2016; Prada et al. 2018; Huang et al. 2025). Rabbit antipredator strategies include acute hearing and eyesight, use of burrows and vegetation cover, physiological responses, an innate capacity to both recognize and discriminate between predators, and well-developed escape behaviors (Villafuerte and Moreno 1997; Pongrácz and Altbäcker 2000; Monclus et al. 2009; Atkins et al. 2016; Martín-Díaz et al. 2018; Alves et al. 2022). Across their distribution, rabbits face highly varied predator regimes, a variety of habitat and other constraints, and in places, persecution by humans as part of pest control programs (Alves et al. 2022; Correa-Cuadros et al. 2023), all of which demand plasticity in their antipredator behavior. Consequently, European rabbits have four prominent influences on their antipredator escape behavior: diurnality and escape (Martín-Díaz et al. 2018; Sogliani et al. 2021), plasticity in vigilance and antipredator responses under different predator regimes (Tobajas et al. 2024), use of cover including adjusting vigilance and escape responses in relation to the availability of cover (Moreno et al. 1996), and sociality (eg, group size) as a mediating influence on predator risk (Villafuerte and Moreno 1997).

In Australia, rabbits quickly became superabundant and a major pest after a successful introduction in 1859 (Alves et al. 2022). They persist in the presence of introduced invasive predators (red fox *Vulpes vulpes* and feral cat *Felis catus*, Abbott 2002; Fairfax 2019), which are historical predators of rabbits within their native range. A handful of locations are fox and/or cat free, due to island isolation, eradication from islands or from safe-havens (eg, predator-proof fenced reserves; Read et al. 2011; Legge et al. 2018; see Table 1). These efforts have effectively created stark contrasts in terrestrial predators to which rabbits are exposed across their range. Rabbits in Australia are active by day and night (Moseby et al. 2005), can detect prevailing predator regimes (Tortosa et al. 2015), move further from cover where there are fewer foxes (Banks et al. 1999), and discriminate their responses between predators (Atkins et al. 2016). However, direct measurements of escape response, by day and night and across predator regimes, are generally absent for rabbits.

**Table 1:** History of European rabbits (*Oryctolagus cuniculus*) and predators at each site sampled in Victoria, Australia.

Predator regime	Site type	Sites (area in ha)	Rabbit colonization	Predator colonization
Cats and foxes	Mainland—no fencing	Cape Liptrap Coastal Park (4,175), Kilcunda Nature Conservation Reserve (53). The Cape, Cape Patterson (30) Cape Patterson Coastal Reserve (45). Warrandyte State Park (680). Wonthaggi Heathland (811). Woodlands Historic Park: outside predator fenced area (820).	*c.* 1860 to 1870 (Williams et al. 1995) for all locations.	Fox = *c.* 1870 to 1890 (Saunders et al. 1995; Abbott et al. 2014) for all locations. Cat = *c.* 1840 to 1850 (Abbott 2002, 2008; Abbott et al. 2014) for all locations.
Cats only	Islands	French Island (17,000). Phillip Island (10,000).	*c.* 1870 (Williams et al. 1995) for both locations.	Cat = *c.* 1840 (Abbott 2002, 2008) for all locations.
Invasive predator-free	Predator-proof fenced reserve	Royal Botanic Gardens, Cranbourne (300).	*c.* 1860 to 1870 for all locations (Williams et al. 1995).	Cats and foxes reduced to low densities by *c.* 2013 (Coates 2021).
		Mt Rothwell Biodiversity Interpretation Center (1,473).		Cats and foxes reduced to low densities by *c.* 2013 (Coates 2021).
		Tiverton conservation farm (800).		Eradicated *c.* 2007.
		Woodlands Historic Park Eastern Barred Bandicoot Sanctuary: predator proof fenced (235).		Eradicated *c.* 2017 with subsequent incursions and management^a^

^a^No known incursions existed at the time of sampling for this study (see Krauss et al. 2025).

Included are the approximate times of invasive colonization or the eradication of cats and foxes.

We compare the nuance in rabbit escape behavior using 4 complementary models: (i) we quantify rabbits’ preferred distance from cover, and how this is influenced by Day/Night, predator regime, group size and starting behavior; (ii) we assess how the probability of rabbits being alert (vigilant) is influenced by Day/Night, predator regime, group size and distance to cover; (iii) we assess how Flight-initiation Distance (FID) varies with all predictors; and (iv) we explore whether the orientation of escape relative to cover differs in relation to all predictors (Fig. 1). FID is the distance at which an animal initiates escape behavior in response to an approaching threat; while the orientation of escape relative to cover specifies whether the animal fled “towards” or “away” from cover. Across all models, we make the following predictions:

1. Although rabbits may use visual, olfactory, or auditory cues to detect an approaching threat, here we assess a visual stimulus (human approaches) without consideration for other modalities, and therefore expect rabbits to maintain shorter distances from cover (Moreno et al. 1996; Villafuerte and Moreno 1997; Banks et al. 1999), have greater vigilance (Monclus and Rödel 2008), longer FIDs (Banks et al. 1999) and to make more escapes towards cover by day than by night (Moreno et al. 1996; Rendall et al. 2024).
2. Where more predators occur, we expect shorter distances to cover, more alertness, longer FIDs and more escapes towards or away from cover relative to where predators are more active by day/night (Moreno et al. 1996; Villafuerte and Moreno 1997; Tortosa et al. 2015; Descalzo Sánchez et al. 2021; Tobajas et al. 2024).
3. We expect proximity to cover will influence alertness with greater alertness in more open areas (Boyce 1984) possibly changing by day versus night (Blanchard et al. 2018), with longer FIDs away from cover (Boyce 1984), and escape oriented towards cover by day but away from cover at night (Moreno et al. 1996; Blanchard et al. 2018).
4. We expected rabbits will be further from cover, exhibit more vigilance, and longer FIDs with increasing group size due to increased detection capacity of larger groups (sensu Stankowich and Blumstein 2005).

**Figure 1:**
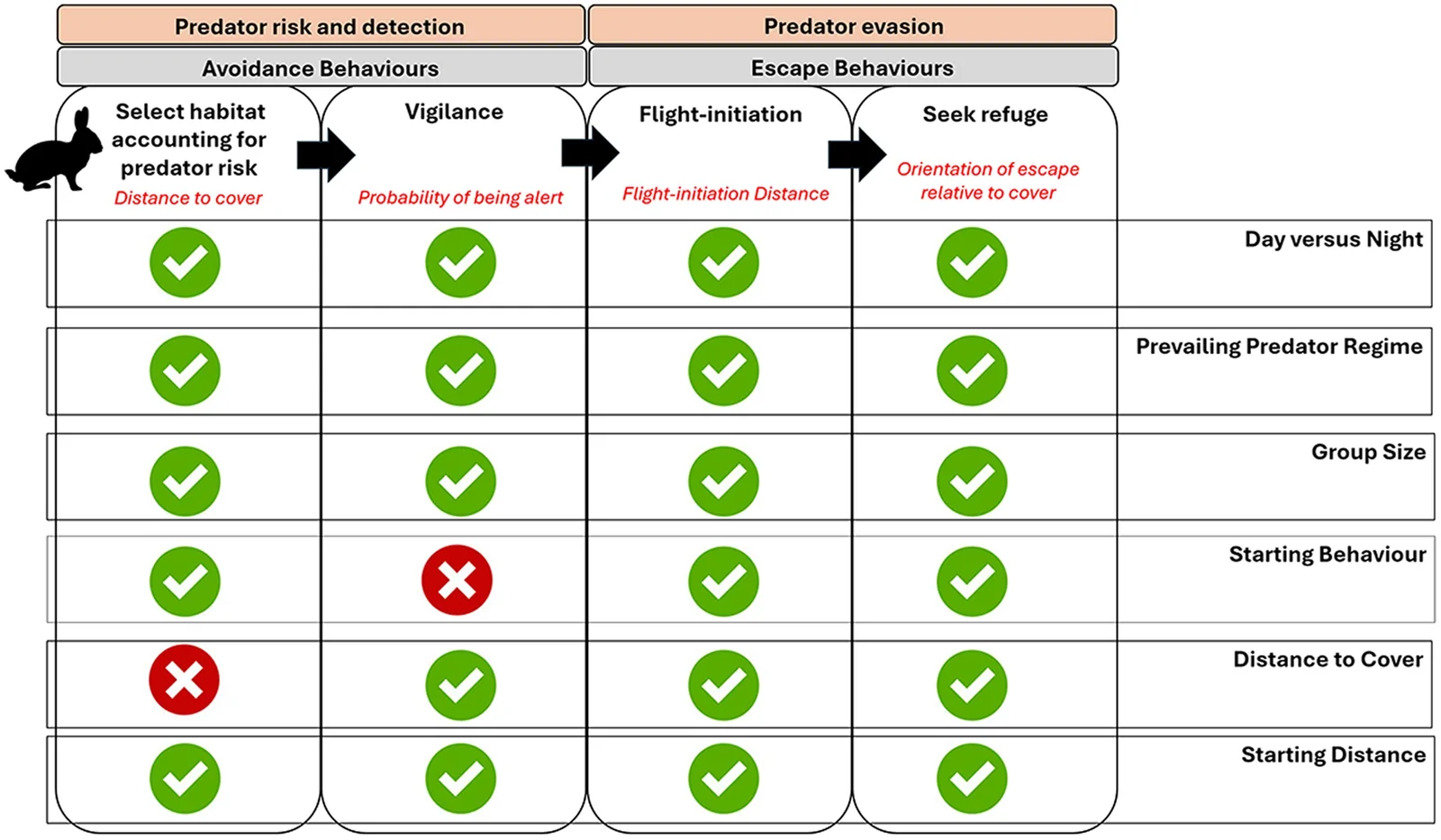
Conceptual diagram of selected predator avoidance behaviors in European rabbits (*Oryctolagus. cuniculus*) (adapted from Tay et al. 2023) demonstrating the focus of our four core models with respect to avoidance and escape behaviors (top). Red italic text shows response variables we measured for each behavior. Ticks represent predictor variables considered for each of our response variables; crosses indicate variables that do not apply.

## Materials and methods

This study was conducted in Victoria, Australia, at sites that represent contrasting invasive predator regimes (Fig. 2; Table 1). Foxes and cats are pervasive and abundant throughout mainland Victoria (Abbott et al. 2014; Legge et al. 2017), and sites without foxes and/or cats are limited to predator-fenced areas and islands on which they have been eradicated or never colonized (Table 1). Rabbits were sampled in areas: (1) with foxes and cats, (2) with cats only, and (3) without cats or foxes. No sites were available where foxes were present, but cats were absent. The predator status of all study sites has been documented (see Krauss et al. 2025), where briefly, mainland sites outside of predator-proof fences have pervasive fox and cat populations. Phillip and French Islands have cats but no foxes thanks to ongoing monitoring and control efforts (Rendall et al. 2021; Eketone et al. 2026; see Fig. 2). While mainland sites within predator proof fenced areas are free of foxes and cats, all sites had diurnal aerial predators of rabbits, in such a way as to be unlikely to confound comparisons (sensu Atkin et al. 2016). All sites featured high densities of rabbits (Roy-Defresne et al. 2019; Alves et al. 2022) and were accessible by day and night. Sampling at these sites occurred both by day (from dawn until early dusk) and night (nighttime) to help control for any site effects (see Figure S1). All sites sampled were outside of urban areas and away from dominant human structures so that this did not confound comparisons between predator regimes.

**Figure 2:**
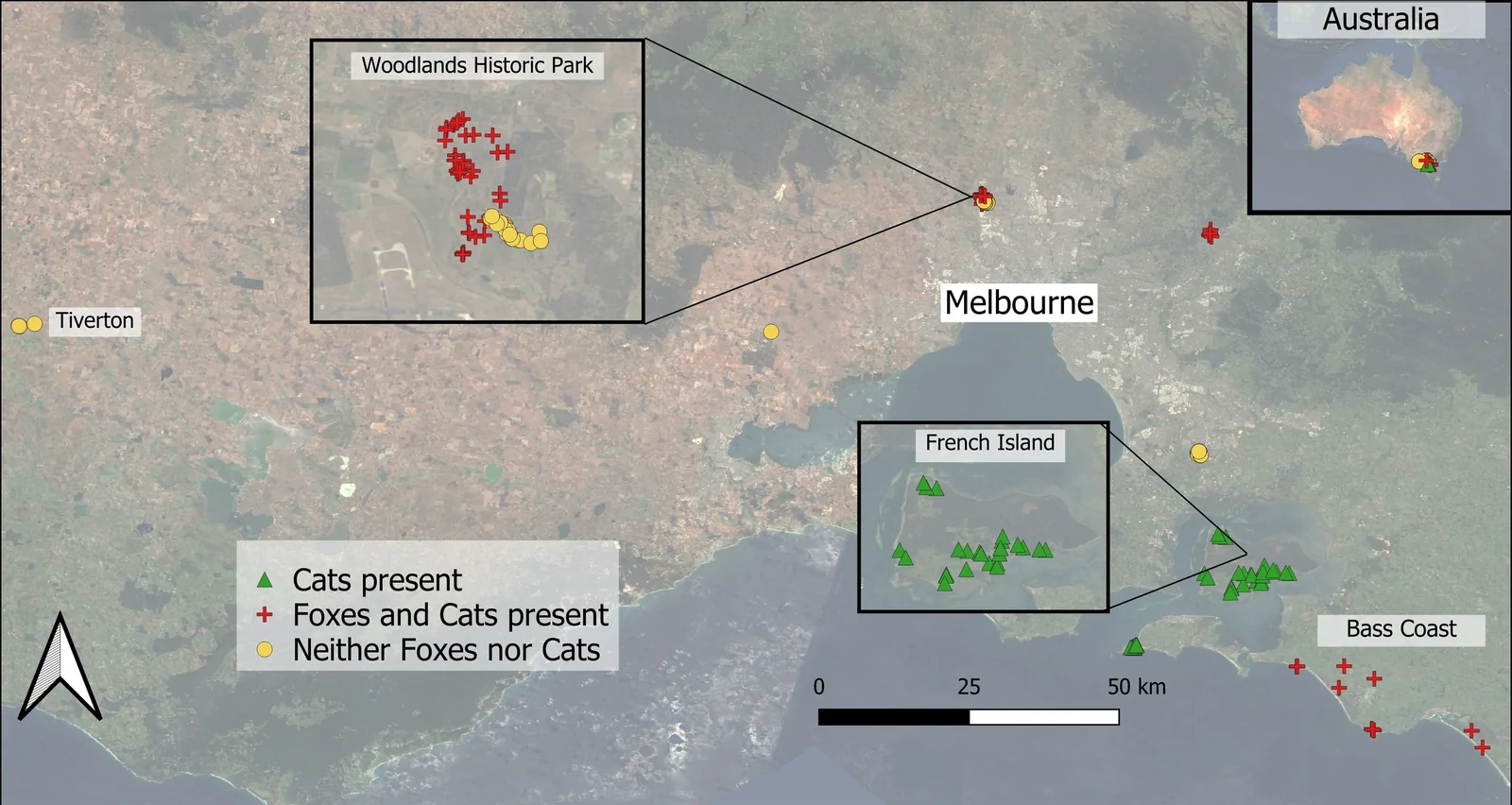
Sampling locations across Victoria, Australia. Green triangles represents areas where only feral cats (*Felis catus*) are present; red crosses represents areas where both red foxes (*Vulpes vulpes*) and cats occur, and yellow circles represents areas where neither feral cats nor red foxes occur. Woodlands Historic Park has a predator free fenced reserve, and surrounding predator present areas.

### Indexing antipredator behavior by day and night

Humans, and possibly prehuman hominids, are historically and currently predators of rabbits (Somerville and Sugiyama 2021), and while rabbits can discriminate their antipredator responses between predators, humans evoke specific and often intense rabbit antipredator behavior (Huang et al. 2025). This study used standardized human approaches towards focal rabbits, which involved recording aspects of rabbit behavior before each approach (Distance to Nearest Cover, Starting Behavior, Starting Distance) and during each approach (Flight-initiation Distance, Modality of Escape and Orientation of Escape relative to cover). Standardized human approaches to wild animals have long been used to index animals’ antipredator behavior (Blumstein 2003) and is being increasingly applied to mammals, with recent technological advancements enabling data collection at night without the use of light (Rendall et al. 2024; Krauss et al. 2025).

Approaches occurred between October and December 2023, with two approaches undertaken in March 2023. This coincided with increasing day lengths in Australia, and we therefore defined a daytime approach as one that occurred when the observer did not require the assistance of a thermal scope; while nighttime approaches required the use of a thermal scope. Daytime approaches generally occurred from early afternoon through to dusk, with nighttime approaches occurring post-dusk into the evening although timing varied with increasing day length throughout sampling (Figure S1). Cloud cover (mean ± SE; Day: 38.6% ± 4.60; Night: 58.92% ± 4.19), temperature (Day: 20.88 °C ± 0.58; Night: 15.10 °C ± 0.57) and wind speed (Day: 5.84 km/h ± 1.27; Night: 6.27 km/h ± 0.99) were reasonably comparable between daytime and nighttime.

All approaches, regardless of time of day, first involved identifying a focal adult rabbit for which there was an uninterrupted view and which had not commenced escape or been previously disturbed by the investigator; where multiple candidate focal individuals were present, one was selected haphazardly. To avoid pseudo-replication, we did not sample within 50 m of a prior observation as humans do not generally elicit an alert response at this distance (after Huang et al. 2025); when we visited a site on repeated nights, we selected distinct nonvisited areas within sites for sampling to avoid pseudo-replication and applied the same 50 m sampling rule where required. Additionally, if any other nonfocal rabbits responded nearby, we avoided sampling them. Moderate degrees of pseudo-replication in studies of mammalian FID are considered unlikely to affect most analyses (Runyan and Blumstein 2004).

Having identified a focal rabbit, we then recorded the focal animal's Group Size, Starting Behavior (foraging, resting or alert), Distance to Nearest Cover (vegetation capable of concealing an adult rabbit), orientation of cover relative to the observer's approach, and distance from the observer at the beginning of an approach (Starting Distance). An individual was deemed alert if it had shown some level of acknowledgement of human presence prior to the approach, for example lifting or turning their head, and/or ear movements in the direction of the approacher. We acknowledge that this may miss some alert responses given physiological responses can precede an attempt to flee (Charuvi et al. 2020). We subsequently analysed whether an individual was alert or not alert (foraging or resting) as a response variable. Orientation of cover was determined using a “clock dial” system whereby an animal fleeing directly away from the observer to cover is deemed fleeing at “12 o’clock” (see Krauss et al. 2025). Group Size was determined by counting all individual rabbits in very close proximity (ie, inter-individual distance of ≤10 m, a distance at which rabbits would interact and often behave cohesively during escape, sensu Huang et al. 2025). Identification of Group Size was comparable between daytime and nighttime. Starting Behavior involved a single classification of “alert”, which could not be reliably resolved to the finer scale of “overt” and “subtle” vigilance described for this species (Monclus and Rödel 2008; Monclus et al. 2009). Modality of Escape (“run” or “walk”) was also recorded but featured little variation and could not be analysed (97% of responses were “run”). All sites where data were collected featured open areas of habitat, typically grassed areas, and offered unimpeded views of focal animals.

The focal animal was then approached directly by a walker at a constant speed (∼1 m/s) until the animal commenced escape, at which point the distance to the animal's initial location (Flight-initiation Distance; FID) was recorded. We also recorded the orientation of escape, using the same dial system used to measure the orientation of nearest cover (thus, allowing the subsequent coding of Orientation of Escape ie “towards” or “away” from cover; see below). Procedures followed Rendall et al. (2024), and the thermal monocular did not emit any light of any wavelength, although sexing rabbits proved impossible (some sex and age-related difference in antipredator behavior of rabbits have been documented (Boyce 1984); we control for age by sampling only adults.

All distances were measured using rangefinders or by pacing (daytime, Bushnell 6 × 24 Engage 1300 Laser Rangefinder; nighttime, HIKMICRO Gryphon GH25L thermal monocular, Hangzhou Microimage Software Co., Ltd., China). When pacing, distances were converted to meters using the known pace length of the specific observer; we used pacing especially at short distances (<10 m) as Mikula (2014) found pacing to be equivalent to distances recorded using rangefinders, and the rangefinders often did not display a distance shorter than 10 m. To ensure that all sampling was conducted uniformly, all approachers were trained (eg, Rendall et al. 2024; Krauss et al. 2025), and estimates of FID are reliable between observers (Guay et al. 2013).

### Statistical analysis

We explore the following response variables which described different aspects of rabbit antipredator behavior: Distance to Cover, Alert or not, FID and Orientation of Escape. Although Group Size varies with predator risk in this species (Boyce 1984; Villafuerte and Moreno 1997; Tobajas et al. 2024), we had insufficient variation to enable a separate model. Models featured the following explanatory variables: Starting Distance, Day/Night, Predator Regime, Starting Behavior, Group Size, Distance to Cover, the interactions between Day/Night and Starting Distance, Day/Night and Predator Regime, Day/Night and Start Behavior; Day/Night and Distance to Cover; Distance to Cover and Group Size, and Distance to Cover and Predator Regime. A random effect of sampling date (ie, day of sampling) nested within location (geographical area within which the predator regime remained constant) was included to account for any variation between sampling days and locations. Models of Distance to Cover excluded distance to nearest cover as a predictor; models of Alert or not excluded the Starting Behavior term (Fig. 1). We were unable to include an interaction between Day/Night and group size as 90% of approaches were to singletons. Predictors were tested for collinearity, and no substantive correlations were present. All possible combinations of predictor variables were considered, including a null model, and model selection performed with Akaike's Information Criterion corrected for small sample sizes (AICc); models with a ΔAICc < 2 were considered to have equal support. Models were validated through simulated residuals plotted against fitted values, and residual values plotted between each predictor variable in the model (Hartig 2022).

To determine the role of distance to cover we modeled how Distance to cover was influenced by our predictor variables listed above. We conducted a log_10_ transformation to normalize Distance to Cover and therefore ran Linear Mixed Models with a Gaussian distribution with model selection and validation performed as above.

To quantify differences associated with the alert status of rabbits prior to the approach, we used the binary response variable (alert or not alert [a combination of foraging and resting]) in a Generalized Linear Mixed Model specifying a binomial distribution. Due to a smaller number of “alert” individuals, we were unable to fit complex models; and several variables were rank deficient (Predator Regime, Foxes and Cats = 3, Cats only = 17, Neither Cats nor Foxes = 1; DayNight, Day = 18; Night = 3). Starting Behavior was correlated with Alert responses and was omitted. We therefore assessed the influence of Starting Distance and Distance from Cover. All model assessment procedures remained the same.

Our models of FID included all terms and procedures listed above. Poisson generalized Linear Mixed Models (GLMM) were initially fitted; however, the response variable was overdispersed. We therefore fit these models with a Negative Binomial distribution which accounts for overdispersion (Zuur et al. 2013).

We coded the Orientation of Escape as towards cover (a movement which was within one graduation either side of the orientation of nearest cover; see Krauss et al. 2025), or else away from cover. GLMM with a binomial distribution were run.

All statistical analyses were run in R 4.2.1 (R Core Team 2023) using the packages “lme4” (Bates et al. 2015), “MuMIn” (Bartoń 2023) for model selection, and “emmeans” (Lenth 2024) to obtain estimated marginal means (EMMs), and “DHARMa” for model validation (Hartig 2022).

## Results

We conducted 172 FIDs by day (*n* = 75) and night (*n* = 97) and across predator regimes: fox and cat present (*n* = 48), cats only (*n* = 60) and neither cat nor fox present (*n* = 64). Overall, Starting Distances were 56.94 ± 3.26 m SE, and FID was 30.37 ± 1.83 m SE. Rabbits were 10.35 ± 1.35 m SE from cover, with Rabbits alert in 12% of responses. Almost all escapes involved “running” (97%; the remainder involved slower escapes ie “walking”). Rabbit escaped towards cover 56% of the time, and away from cover 44% of the time.

The distance the rabbit was from cover was best supported by a model featuring Starting Distance and Day/Night (AIC *ώ* = 0.326, Table 2; Table S2). There was also support for the influence of Starting Behavior (ΔAIC = 0.72). Distance to Cover increased with increasing Starting Distance (*β* = 0.125, 95%CI: 0.057 to 0.193; Fig. 3). Rabbits were further from cover at nighttime (*µ* = 7.25, 95%CI: 5.29 to 9.83) compared with daytime (*µ* = 2.70, 95%CI: 1.74 to 4.01) but were closer to cover when more vigilant (*µ* = 1.28, 0.23 to 3.24) compared with when foraging or resting (Foraging, *µ* = 6.72, 4.37 to 10.10; Resting, *µ* = 4.75, 2.99 to 7.31; Fig. 3); no difference was observed between foraging or resting (*P* > 0.05).

**Figure 3:**
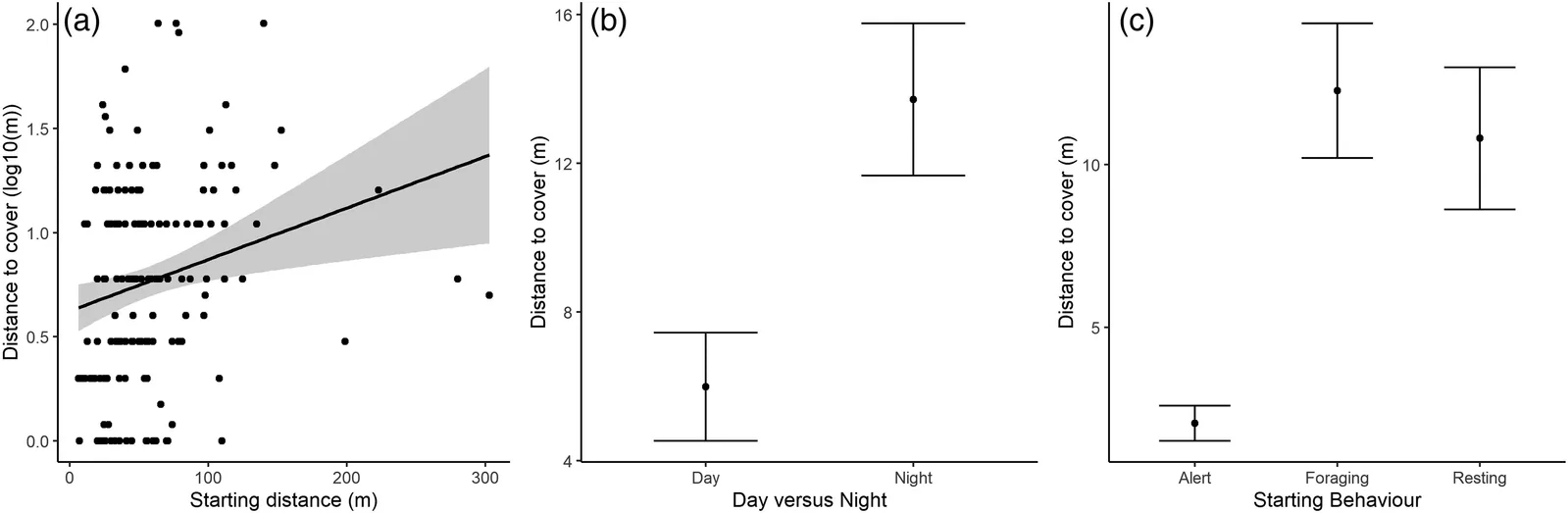
The relationship between European Rabbit (*Oryctolagus cuniculus*) distance to cover and Starting Distance a), day versus nighttime b) and Starting Behavior c) (*µ* ± 95% CI).

**Table 2:** Model selection table exploring the effect of Starting Distance (SD), day versus night (DN), group size (GroupSize) and Starting Behavior (StartBeh) on Distance to Cover (DC) and Alertness (A), Flight Initiation Distances (FIDs), and orientation of escape (Flight Towards Cover; FTC) of European rabbits *O. cuniculus*.

Model	Model structure	*K*	LL	AICc	ΔAICc	AICc *ω*	*R* ^2^_m_	*R* ^2^_c_
Distance to cover	DC ∼ SD + DN	6	−111.882	236.3	0.00	0.326	0.168	0.168
	DC ∼ DN + StartBeh	7	−111.157	237.0	0.72	0.227	0.202	0.268
	DC ∼ SD + DN + StartBeh	8	−110.186	237.3	0.98	0.200	0.220	0.261
	DC ∼ SD + DN + StartBeh + DN × SD	9	−110.500	240.1	3.84	0.048	0.234	0.287
	DC ∼ 1	4	−123.373	255.0	18.71	0.000	0.000	0.000
Alert	A ∼ SD + DC	5	−31.662	73.7	0.00	0.752	0.628	0.758
	A ∼ DC	4	−33.830	75.9	2.21	0.248	0.630	0.740
	A ∼ SD	4	−42.161	92.6	18.88	0.000	0.026	0.330
	A ∼ 1	3	−44.227	94.6	35.69	0.000	0.000	0.000
FID	FID ∼ SD	5	−685.181	1380.7	0.00	0.293	0.409	0.409
	FID ∼ SD + DN	6	−684.901	1382.3	1.59	0.132	0.411	0.411
	FID ∼ SD + GroupSize	6	−684.940	1382.4	1.66	0.127	0.412	0.412
	FID ∼ SD + StartBeh	7	−684.300	1383.3	2.56	0.081	0.415	0.415
	FID ∼ 1	4	−743.097	1494.4	113.71	0.000	0.000	0.000
Flight towards cover	FTC ∼ DN + DC + DN × DC	6	−98.365	209.3	0.00	0.094	0.058	0.058
	FTC ∼ DN + DC + DN × DC + PreReg + PreReg × DC	10	−93.930	209.4	0.11	0.089	0.120	0.120
	FTC ∼ DN	4	−101.515	211.3	1.99	0.035	0.017	0.017
	FTC ∼ DN + DC	5	−100.447	211.3	1.99	0.035	0.031	0.031
	FTC ∼ DN + DC + DN × DC + PreReg	8	−97.209	211.4	2.11	0.033	0.074	0.074
	FTC ∼ 1	3	−102.765	211.7	2.38	0.029	0.000	0.000

*K* is number of parameters; LL is Log Likelihood. Models shown are those with ΔAICc ≤ 2, the next best model for context, and the intercept only model. All models are presented in Supplementary material.

Rabbits being alert prior to the initiation of an approach was related to both the Starting Distance and Distance to Cover (AIC *ώ* = 0.628). The probability of a rabbit being Alert declined with increasing Starting Distance (*β* = −0.703, 95% CI: 0.098 to −1.504) and Distance to Cover (*β* = −5.161, 95% CI: −1.312 to −9.010) although confidence intervals overlapped zero for Starting Distance (Fig. 4).

**Figure 4:**
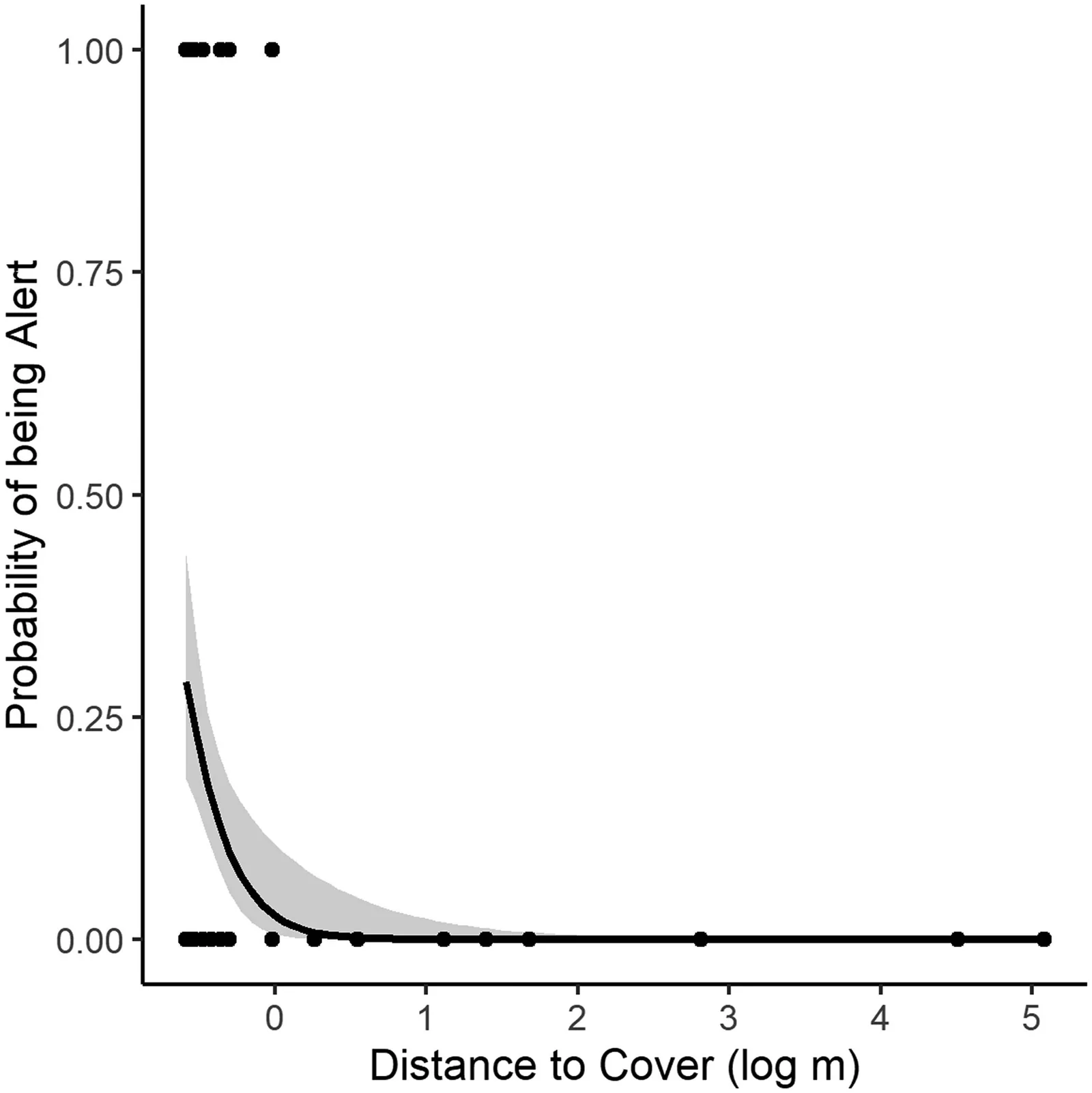
Relationship between the probability of a European Rabbit (*Oryctolagus cuniculus*) being Alert and Distance to cover (logged).

Starting Distance was the only predictor variable in the top model of FID (AICc *ώ* = 0.277; Table 2, Table S1). Day/Night (ΔAICc = 1.59) and Group Size (ΔAICc = 1.66) also received some support. There was no support for an effect of predator regime (ΔAICc = 3.73), the interaction between Starting Distance and Day/Night (ΔAICc = 2.99), predator regime and Day/Night (ΔAICc = 8.26), and Starting Behavior and Day/Night (ΔAIC = 8.27). FID of rabbits increased with longer Starting Distances (*β* = 0.421, 95%CI: 0.333 to 0.510; Fig. 5). FIDs were marginally longer by day (Mean = 28.5 m, 95%CI: 25.3 to 32.1) than night (27.1 m, 95%CI: 24.5 to 30.0); and FIDs apparently increased with increasing group size (*β* = 0.027, 95%CI: −0.160 to 0.213), but confidence intervals substantially crossed zero and these likely reflect uninformative parameters.

**Figure 5:**
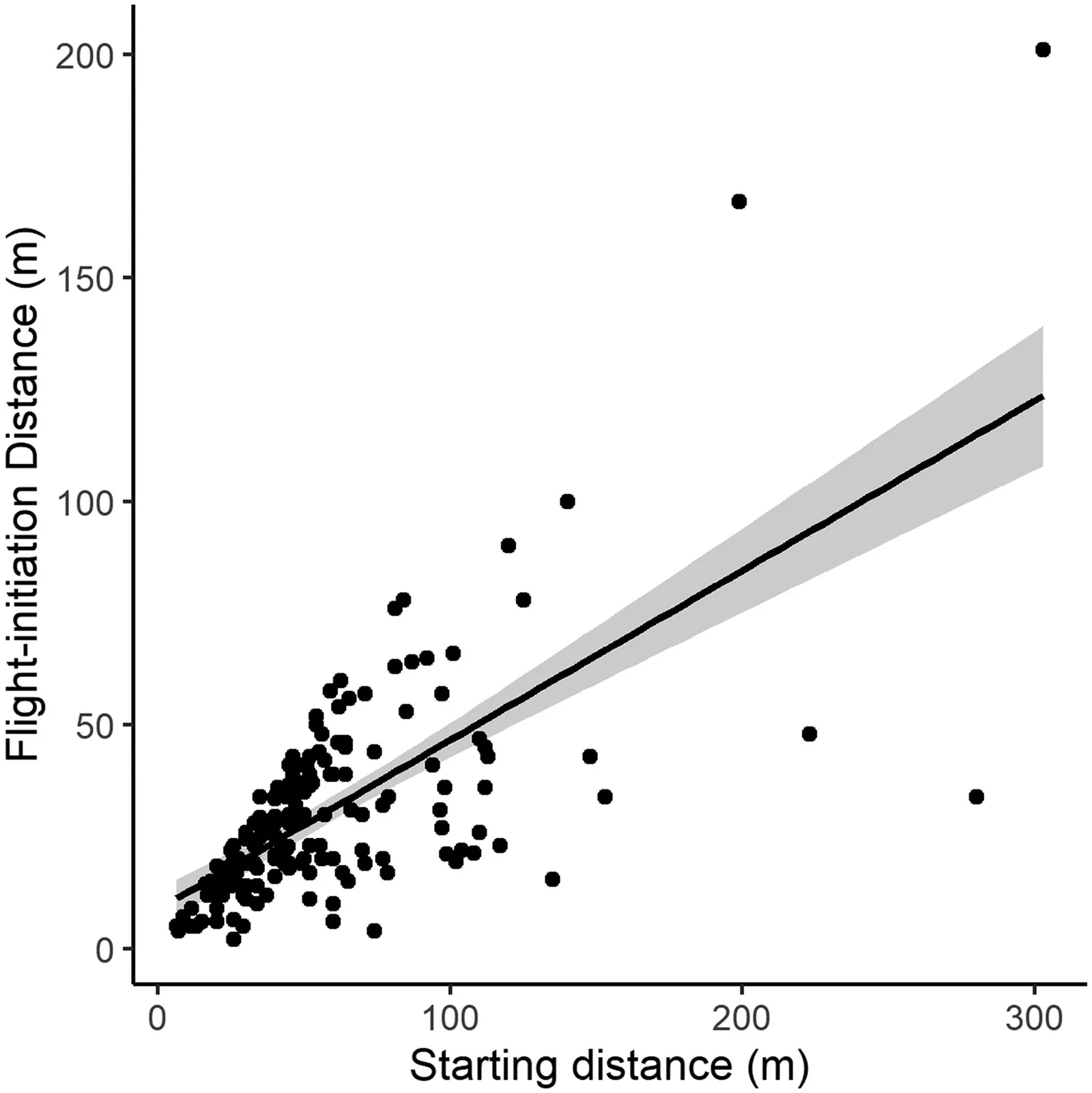
Relationship between Flight-initiation Distance and Starting Distance for European rabbits (*Oryctolagus cuniculus*).

The probability of rabbits fleeing towards cover was influenced by Distance to Cover, whether it was daytime or nighttime and the interaction between these (AIC *ώ* = 0.094; Table 2; Table S3). There was also support for an influence of Predator Regime and its interaction with Distance to Cover (ΔAICc = 0.11). Rabbits fled more directly towards cover the further they were from cover at nighttime but there was no trend during daytime (Fig. 6). Rabbits also fled more directly to cover the further from cover when they were in the absence of predators, however, there was no trend when they were in the presence of either cats, or foxes and cats (Fig. 7).

**Figure 6:**
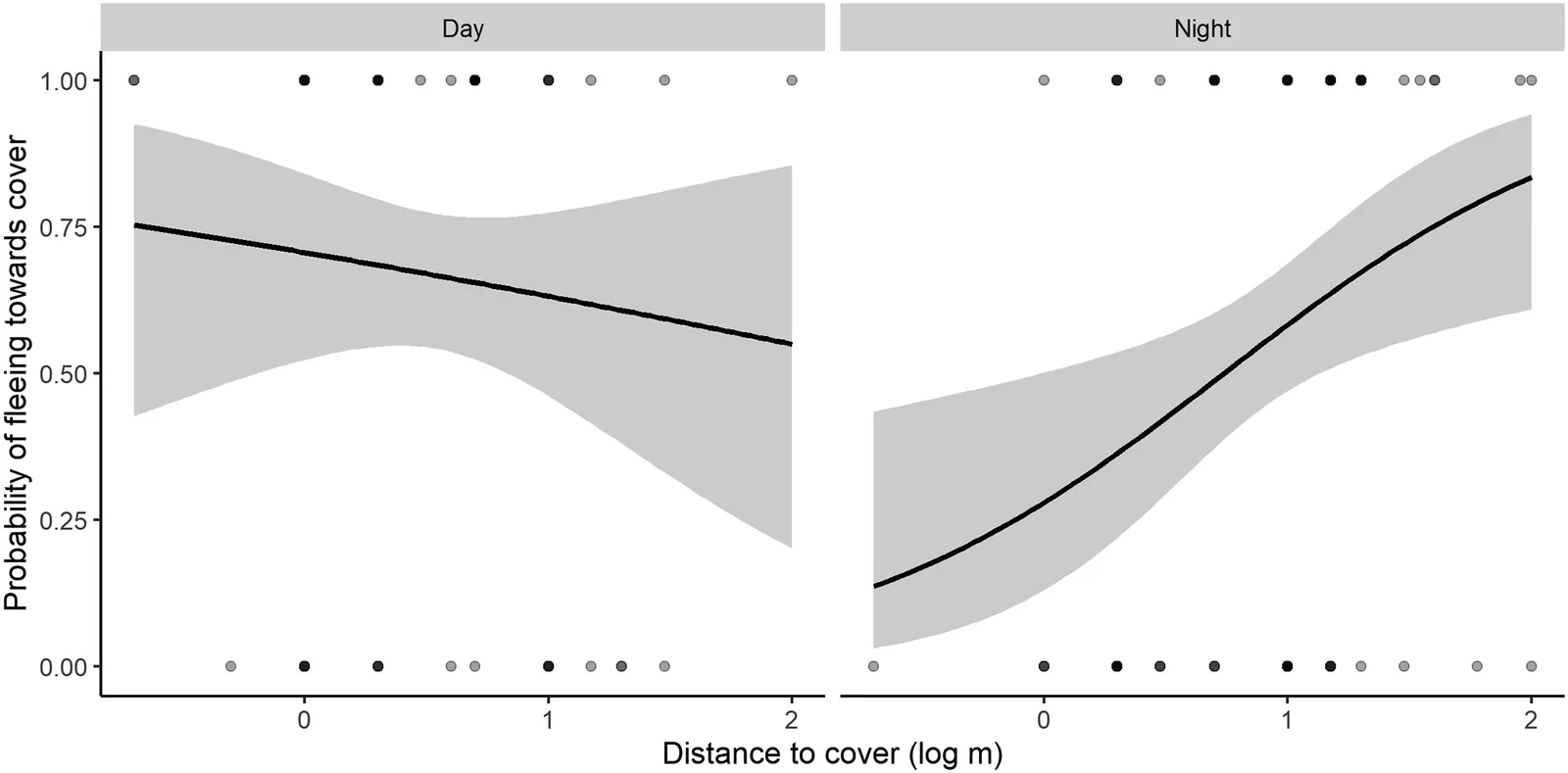
The probability of a European rabbit (*Oryctolagus cuniculus*) fleeing towards cover (estimate ± 95% CI) in relation to distance to cover (log m) by day and by night.

**Figure 7:**
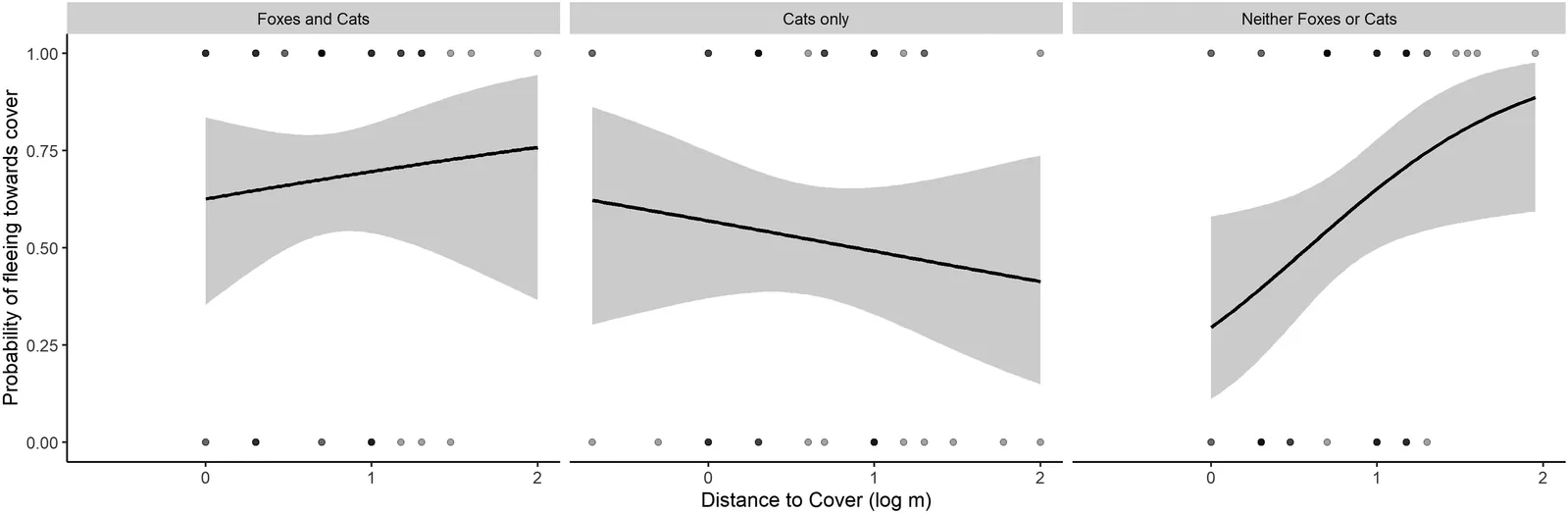
The probability of a European rabbit (*Oryctolagus cuniculus*) fleeing towards cover (estimate ± 95% CI) in relation to Distance to Cover (log m) across three different predator regimes.

## Discussion

Cathemeral species face the challenge of escaping predators during two starkly different periods, potentially requiring different sensory modalities. Here we show that some, but not all behaviors differ between daytime and nighttime; rabbits’ use of cover changed, while the timing of escape was consistent suggesting that their sensory capacity to detect approaching threats is at least as effective by night as it is by day. FIDs can reveal specific escape tactics of animals (eg, Sanchez-Gomez et al. 2024). The use of specific nocturnal escape techniques (eg, a sit and run strategy which may be more effective by night) was not evident in European rabbits but may be present in other species. Given the prominence of nocturnal predators for many prey species, it is unsurprising that prey have evolved and/or adapted effective nighttime antipredator strategies (Gaston 2019).

We found that rabbits moved further from cover at night, as has previously been recorded in Australia (Banks et al. 1999) and elsewhere for this species (eg, Kerguelen archipelago, Blanchard et al. 2018). We show that this effect was not influenced by the presence of mammalian predators (mostly nocturnal, ambush and therefore cover-using predators; but see Banks et al. 1999 and Tobajas et al. 2024). This suggests that the risk from diurnal raptors, key predators of rabbits in open Australian habitats by day (Robley et al. 2004) and which occurred across all study sites, may be driving this pattern in our study system. Forage quality may be higher further from cover (but see Banks et al. 1999 and Bakker et al. 2005), and predator risk near cover may be higher at night (Moreno et al. 1996; Blanchard et al. 2018), making nocturnal use of patches away from cover more profitable. Such tradeoffs between foraging and antipredator behavior occur in rabbits and in at least some other lagomorphs (Bakker et al. 2005; Monclus and Rodel 2008; Lagos et al. 2009). We acknowledge that other factors, unmeasured by us, may also influence rabbit nocturnal movements and therefore proximity to cover (eg moon phase; Penteriani et al. 2013).

Maintaining and adjusting proximity to cover is a well-established antipredator risk strategy in rabbits (Monclus et al. 2009; Tobajas et al. 2024), including in Australia (Banks et al. 1999). Distance to cover increased with Starting Distance and during the night, with rabbit alertness decreasing the further they were from cover. The somewhat counterintuitive finding that alertness is greatest nearer cover, by day and night, may reflect cover being a possible source of ambush predators (eg, cats and foxes; Moreno et al. 1996; Blanchard et al. 2018; Tobajas et al. 2024), reduced viewshed, recent emergence of focal rabbits from cover, or inter-individual variation in risk-taking (eg, individuals may vary in their capacity to avoid predators, Penteriani et al. 2008, or young may remain closer to cover, Boyce 1984). We approached only adults; however, the capacity to differentiate fully grown sub-adults, especially by night, is a possible limitation of our study. Distance to cover increased with Starting Distance, raising the untested possibility of undetected preapproach responses which saw rabbits approach cover before we detected them. However, shorter Starting Distances also likely occurred in slightly more closed habitat situations where prevailing cover was already closer, suggesting this finding could be artefactual in nature.

The probability of a rabbit being alert increased closer to cover (see above) but did not vary with day/night (but see Blanchard et al. 2018), or with group size or predator regime as is often reported and was expected (Boyce 1984; Monclus and Rodel 2008; Tobajas et al. 2024). Rabbits on the Kerguelen Islands increased vigilance near cover at night, presumably to manage risk posed by cover-using predatory cats (Blanchard et al. 2018). Vigilance in rabbits serves both social and antipredator functions, and varies with age, body condition, social status and, for females, pregnancy status (Monclus and Rodel 2008)—factors which we could not measure (especially at night). The role of vigilance in sociality (Monclus and Rödel 2008; Monclus et al. 2009) may at least partly explain the lack of an effect of predator regime in this study. The lack of difference in alertness (and modest differences in FIDs) between day and night and the positive relationship between Starting Distance and FID suggests that the sensory capacity to detect approaching threats is at least as effective by night as by day. Standardized walker approaches to mammals often feature alert individuals by day and night (Rendall et al. 2024), perhaps because rabbits may be able to detect a human at distances which exceed those at which the human detects the focal animal (Plotz and Linklater 2020). Detailed assessments of detection distances of mammals at night may lead to methodological refinements, or improvements in interpretation of alertness of focal animals when discovered, when using standardized approaches to evoke antipredator responses.

We report marginally longer FIDs by day. Given the prominence of nocturnally active predators for many prey, it is unsurprising that prey have evolved and/or adapted effective nighttime antipredator strategies (eg, Schulke 2001; Stankowich et al. 2014). In many ecosystems rabbits persist under intense predator pressure (Martín-Díaz et al. 2018). We expected longer FIDs where predators occurred, yet this did not occur. Rabbits have a long evolutionary coexistence with eutherian predators (Martín-Díaz et al. 2018) which in Australia continue to prey on rabbits heavily (Doherty et al. 2015; Stobo-Wilson et al. 2022) along with diurnal native predators (Robley et al. 2004). Rabbits are declared pests in Australia and therefore actively controlled by humans across our predator gradient (Strive and Cox 2019); all areas and times we sampled might conceivably feature high perceived predator risk for rabbits. European rabbits with limited predator exposure may initially be more responsive to perceived predator risk with responses quickly attenuating thereafter (Tortosa et al. 2015; Prada et al. 2018), potentially reducing any differences in FID between prevailing predator regimes.

Rabbit escape by day does not always end in refuge (cover or a burrow; Ziege et al. 2016), and we found this to be also true by night. The orientation of escape relative to cover is plastic and varies with context (ie, urbanization) in rabbits (Ziege et al. 2016). Rabbits fled more directly towards cover the further they were from cover at night (but not day), and only in the absence of cats and foxes but not in other prevailing predator regimes. Cover may represent safety by day (protection from avian predators) but greater risk by night (increased risk of terrestrial ambush predators—foxes and cats). This has been shown previously for rabbits (Moreno et al. 1996; Blanchard et al. 2018) and is consistent with our finding that escape towards cover occurs only where nocturnal, mammalian ambush predators do not occur. Thus, the use of cover and the concept of refuge may vary by day and night and in relation to prevailing predator regimes (ie between sites).

Although few data are available, we report FIDs of introduced rabbits that were remarkably similar to those recorded from human approaches within the species’ original natural range (ie, within 50 km from where Australia's rabbits were originally sourced; Huang et al. 2025; Table S4). FIDs were longer with longer SDs; the positive relationship between Starting Distance and FID is commonly reported across a variety of taxa including mammals during the day and at night (Blumstein 2003; Brubaker and Coss 2016; Rendall et al. 2024). Starting Distance has been interpreted as having functional significance (leave early and avoid the rush’; Blumstein 2003) but also may be at least partly a mathematical artifact (it must always exceed FID; Dumont et al. 2012). We note that rabbit FIDs can vary in relation to their life history stage and the nature of the approaching stimulus (Table S4; Atkens et al. 2016), aspects that we could not measure or did not test, especially by night.

Studies of nocturnal antipredator behavior remain rare; for a cathemeral species our multimetric study shows complex rabbit antipredator behavior which is consistent or varies in relation to contextual factors including daytime and nighttime. The similarity in escape responses (FID) observed here suggests that for rabbits, the capacity to detect a predator may be similar by day and night. Contrary to our prediction, this did not vary with predator regime although it has been observed for endemic threatened mammals in Australia (see Krauss et al. 2025). Our results emphasize how nighttime can influence antipredator behaviors in unique ways that don’t always align with hypotheses that are often based on daytime research. With the general neglect of nighttime ecology, we encourage more studies of escape ecology to include nighttime comparisons.

## Supplementary Material

arag123_Supplementary_Data

## Data Availability

Analyses reported in this article can be reproduced using the data provided by Di Stefano et al. (2026).
