# Supplementary material for: Antipredator behavior of European rabbits by day and night
Source: Behav Ecol. 2026 Oct 16;37(6):arag123. doi: 10.1093/beheco/arag123 (PMC13687647; doi:10.1093/beheco/arag123)
Supplement: Supplementary file 1 [file arag123_supplementary_data.docx]

**Supplementary Material**

**Antipredator behaviour of European rabbits by day and night**

**Figure S1.** The distribution of times where daytime and nighttime Flight-initiation distances (FIDs) were conducted. The “density” refers to the relative frequency of FIDs within a particular time period, with the solid black line representing daytime FIDs and the dashed line representing nighttime FIDs. The overlap in time period observed is due to sampling occurring between October and December in Australia where day length is progressively getting longer, and ‘daylight savings’.

**Table S1.** Model selection table for Rabbit FIDs including all models with a cumulative Akiake’s Information Criterion weighting of 0.95. Variables include: Flight-initiation Distance (FID), Starting Distance (SD), Day/Night (DN), Group Size (GS), Distance to Cover (Dcover), Starting Behaviour (SBeh), Predator Regime (PredReg). Reported parameters include the number of parameters in the model (K), Loglikelihood (LL), Akiake Information Criterion corrected (AICc), delta AICc (ΔAICc) and Akiake’s weights (AICc ώ).

| **Model** | **K** | **LL** | **AICc** | **ΔAICc** | **AICc ώ** |
| --- | --- | --- | --- | --- | --- |
| FID ~ SD | 5 | -685.308 | 1381.0 | 0.00 | 0.184 |
| FID ~ SD + DN | 6 | -685.020 | 1382.5 | 1.57 | 0.084 |
| FID ~ SD + GS | 6 | -685.061 | 1382.6 | 1.65 | 0.080 |
| FID ~ SD + Dcover | 6 | -685.283 | 1383.1 | 2.10 | 0.064 |
| FID ~ SD + SBeh | 7 | -684.405 | 1383.5 | 2.52 | 0.052 |
| FID ~ SD + DN + DN*PredReg | 7 | -684.625 | 1383.9 | 2.96 | 0.042 |
| FID ~ SD + Dcover + GS + Dcover*GS | 8 | -683.630 | 1384.1 | 3.17 | 0.038 |
| FID ~ SD + DN + GS | 7 | -684.810 | 1384.3 | 3.32 | 0.035 |
| FID ~ SD + Dcover + DN | 7 | -684.938 | 1384.6 | 3.58 | 0.031 |
| FID ~ SD + PredReg | 7 | -684.998 | 1384.7 | 3.70 | 0.029 |
| FID ~ SD + Dcover + GS | 7 | -685.039 | 1384.8 | 3.78 | 0.028 |
| FID ~ SD + GS + SBeh | 8 | -684.200 | 1385.3 | 4.31 | 0.021 |
| FID ~ SD + DN + SBeh | 8 | -684.326 | 1385.5 | 4.56 | 0.019 |
| FID ~ SD + Dcover + SBeh | 8 | -684.334 | 1385.6 | 4.57 | 0.019 |
| FID ~ SD + DN + GS + DN*PredReg | 8 | -684.454 | 1385.8 | 4.81 | 0.017 |
| FID ~ SD + Dcover + DN + GS + Dcover*GS | 9 | -683.379 | 1385.9 | 4.89 | 0.016 |
| FID ~ SD + DN + PredReg | 8 | -684.576 | 1386.0 | 5.06 | 0.015 |
| FID ~ SD + Dcover + DN + DN*PredReg | 8 | -684.607 | 1386.1 | 5.12 | 0.014 |
| FID ~ SD + PredReg + SBeh | 9 | -683.523 | 1386.2 | 5.18 | 0.014 |
| FID ~ SD + GS + PredReg | 8 | -684.664 | 1386.2 | 5.23 | 0.013 |
| FID ~ SD + Dcover + DN + GS | 8 | -684.735 | 1386.4 | 5.38 | 0.012 |
| FID ~ SD + Dcover + DN + Dcover*DN | 8 | -684.896 | 1386.7 | 5.70 | 0.011 |
| FID ~ SD + Dcover + PredReg | 8 | -684.971 | 1386.8 | 5.85 | 0.010 |
| FID ~ SD + Dcover + GS + SBeh + Dcover*GS | 10 | -682.877 | 1387.1 | 6.14 | 0.009 |
| FID ~ SD + DN + SBeh + DN*PredReg | 9 | -684.008 | 1387.1 | 6.15 | 0.008 |
| FID ~ SD + DN + PredReg + DN*PredReg | 9 | -684.062 | 1387.2 | 6.26 | 0.008 |
| FID ~ SD + DN + GS + SBeh | 9 | -684.132 | 1387.4 | 6.40 | 0.007 |
| FID ~ SD + Dcover + GS + SBeh | 9 | -684.139 | 1387.4 | 6.41 | 0.007 |
| FID ~ SD + Dcover + DN + SBeh | 9 | -684.220 | 1387.6 | 6.57 | 0.007 |
| FID ~ SD + Dcover + DN + GS + Dcover*GS + DN*PredReg | 10 | -683.101 | 1387.6 | 6.59 | 0.007 |
| FID ~ SD + Dcover + GS + PredReg + Dcover*GS | 10 | -683.116 | 1387.6 | 6.62 | 0.007 |
| FID ~ SD + DN + GS + PredReg | 9 | -684.283 | 1387.7 | 6.70 | 0.006 |
| FID ~ SD + GS + PredReg + SBeh | 10 | -683.234 | 1387.8 | 6.86 | 0.006 |
| FID ~ SD + Dcover + DN + GS + DN*PredReg | 9 | -684.437 | 1388.0 | 7.01 | 0.006 |
| FID ~ SD + Dcover + PredReg + SBeh | 10 | -683.335 | 1388.0 | 7.06 | 0.005 |
| FID ~ SD + Dcover + DN + PredReg | 9 | -684.475 | 1388.1 | 7.08 | 0.005 |
| FID ~ SD + Dcover + DN + Dcover*DN + DN*PredReg | 9 | -684.503 | 1388.1 | 7.14 | 0.005 |
| FID ~ SD + Dcover + DN + GS + Dcover*DN + Dcover*GS | 10 | -683.378 | 1388.1 | 7.14 | 0.005 |
| FID ~ SD + DN + PredReg + SBeh | 10 | -683.475 | 1388.3 | 7.34 | 0.005 |
| FID ~ SD + Dcover + GS + PredReg | 9 | -684.637 | 1388.4 | 7.41 | 0.005 |
| FID ~ SD + Dcover + DN + GS +Dcover*DN | 9 | -684.676 | 1388.5 | 7.48 | 0.004 |
| FID ~ SD + DN + GS + PredReg + DN*PredReg | 10 | -683.824 | 1389.0 | 8.04 | 0.003 |
| FID ~ SD + Dcover + DN + GS + PredReg + Dcover*GS | 11 | -682.709 | 1389.1 | 8.09 | 0.003 |
| FID ~ SD + DN + GS + SBeh + DN*PredReg | 10 | -683.854 | 1389.1 | 8.10 | 0.003 |
| FID ~ SD + DN + PredReg + DN*PredReg | 10 | -683.896 | 1389.2 | 8.18 | 0.003 |
| FID ~ SD + DN + SBeh | 10 | -683.908 | 1389.2 | 8.20 | 0.003 |
| FID ~ SD + Dcover + DN + GS + SBeh + Dcover*GS | 11 | -682.798 | 1389.2 | 8.27 | 0.003 |
| FID ~ SD + Dcover + GS + PredReg + SBeh + Dcover*GS | 12 | -681.671 | 1389.3 | 8.33 | 0.003 |
| FID ~ SD + Dcover + DN + SBeh + DN*PredReg | 10 | -683.970 | 1389.3 | 8.33 | 0.003 |
| FID ~ SD + Dcover + DN + GS + SBeh | 10 | -684.040 | 1389.4 | 8.47 | 0.003 |
| FID ~ SD + Dcover + DN + PredReg + DN*PredReg | 10 | -684.045 | 1389.5 | 8.48 | 0.003 |
| FID ~ SD + Dcover + DN + SBeh + Dcover*DN | 10 | -684.138 | 1389.6 | 8.66 | 0.002 |
| FID ~ SD + Dcover + DN + GS + PredReg | 10 | -684.188 | 1389.7 | 8.76 | 0.002 |
| FID ~ SD + Dcover + GS + PredReg + SBeh | 11 | -683.062 | 1389.8 | 8.80 | 0.002 |
| FID ~ SD + Dcover + DN + GS + Dcover*DN + Dcover*GS + DN*PredReg | 11 | -683.096 | 1389.8 | 8.86 | 0.002 |
| FID ~ SD + Dcover + DN + GS + Dcover*DN + DN*PredReg | 10 | -684.311 | 1390.0 | 9.01 | 0.002 |

**Table S2**. Model selection table for Rabbit FIDs including all models with a cumulative Akiake’s Information Criterion weighting of 0.95. Variables include: Flight-initiation Distance (FID), Starting Distance (SD), Day/Night (DN), Group Size (GS), Distance to Cover (Dcover), Starting Behaviour (SBeh), Predator Regime (PredReg). Reported parameters include the number of parameters in the model (K), Loglikelihood (LL), Akiake Information Criterion corrected (AICc), delta AICc (ΔAICc) and Akiake’s weights (AICc ώ).

| **Model** | **K** | **LL** | **AICc** | **ΔAICc** | **AICc ώ** |
| --- | --- | --- | --- | --- | --- |
| DC ~ SD + DN | 6 | -111.882 | 236.3 | 0.00 | 0.344 |
| DC ~ DN + SBeh | 7 | -111.157 | 237.0 | 0.72 | 0.240 |
| DC ~ SD + DN + SBeh | 8 | -110.186 | 237.3 | 0.98 | 0.211 |
| DC ~ SD + DN + SBeh + DN*SD | 9 | -110.500 | 240.1 | 3.84 | 0.051 |
| DC ~ SD + DN + DN*SD | 7 | -112.716 | 240.1 | 3.84 | 0.050 |
| DC ~ SBeh | 6 | -114.295 | 241.1 | 4.83 | 0.031 |
| DC ~ DN | 5 | -115.477 | 241.3 | 5.04 | 0.028 |
| DC ~ SD + DN + GS | 7 | -114.011 | 242.7 | 6.43 | 0.014 |
| DC ~ DN + SBeh + GS | 8 | -112.994 | 242.9 | 6.60 | 0.013 |
| DC ~ DN + SBeh | 9 | -112.123 | 243.4 | 7.09 | 0.010 |
| DC ~ SD + SBeh | 7 | -114.363 | 243.4 | 7.14 | 0.010 |

**Table S3.** Model selection table for whether rabbits fled towards cover including all models with a cumulative Akiake’s Information Criterion weighting of 0.95. Variables include: Starting Distance (SD), Day/Night (DN), Group Size (GS), Distance to Cover (DC), Starting Behaviour (SB), Predator Regime (PR). Reported parameters include the number of parameters in the model (K), Loglikelihood (LL), Akiake Information Criterion corrected (AICc), delta AICc (ΔAICc) and Akiake’s weights (AICc ώ).

| **Model** | **K** | **LL** | **AICc** | **ΔAICc** | **AICc ώ** |
| --- | --- | --- | --- | --- | --- |
| FTC ~ DC+DN+DC*DN | 6 | -98.365 | 209.3 | 0.00 | 0.094 |
| FTC ~ DC+DN+PR+DC*DN+DC*PR | 10 | -93.930 | 209.4 | 0.11 | 0.089 |
| FTC ~ DN | 4 | -101.515 | 211.3 | 1.99 | 0.035 |
| FTC ~ DC+DN | 5 | -100.447 | 211.3 | 1.99 | 0.035 |
| FTC ~ DC+DN+PR+DC*DN | 8 | -97.209 | 211.4 | 2.11 | 0.033 |
| FTC ~ DC+DN+SD+DC*DN | 7 | -98.353 | 211.5 | 2.17 | 0.032 |
| FTC ~ DC+DN+GS+DC*DN | 7 | -98.365 | 211.5 | 2.20 | 0.031 |
| FTC ~ DC+DN+PR+DC*PR | 9 | -96.198 | 211.7 | 2.35 | 0.029 |
| FTC ~ DC+DN+PR+SD+DC*DN+DC*PR | 11 | -93.896 | 211.7 | 2.37 | 0.029 |
| FTC ~ 1 | 3 | -102.765 | 211.7 | 2.38 | 0.029 |
| FTC ~ DC+DN+GS+PR+DC*DN+DC*PR | 11 | -93.929 | 211.7 | 2.43 | 0.028 |
| FTC ~ DC+DN+PR+DC*DN+DC*PR+DN*PR | 12 | -93.305 | 212.9 | 3.54 | 0.016 |
| FTC ~ DC+DN+SB+DC*DN | 8 | -97.963 | 212.9 | 3.62 | 0.015 |
| FTC ~ PR | 5 | -101.294 | 213.0 | 3.69 | 0.015 |
| FTC ~ DC+DN++PR++SB+DC*DN+DC*PR | 12 | -93.397 | 213.0 | 3.73 | 0.015 |
| FTC ~ DC | 4 | -102.417 | 213.1 | 3.80 | 0.014 |
| FTC ~ DC+DN+PR+DC*DN+DN*PR | 10 | -95.783 | 213.1 | 3.82 | 0.014 |
| FTC ~ DC+PR+DC*PR | 8 | -98.060 | 213.1 | 3.82 | 0.014 |
| FTC ~ DN+PR | 6 | -100.386 | 213.4 | 4.04 | 0.012 |
| FTC ~ DN+SD | 5 | -101.492 | 213.4 | 4.09 | 0.012 |
| FTC ~ DN+GS | 5 | -101.495 | 213.4 | 4.09 | 0.012 |
| FTC ~ DC+DN+SD | 6 | -100.423 | 213.4 | 4.11 | 0.012 |
| FTC ~ DC+DN+GS | 6 | -100.446 | 213.5 | 4.16 | 0.012 |
| FTC ~ DC+DN+PR+SD+DC*PR | 10 | -96.012 | 213.6 | 4.27 | 0.011 |
| FTC ~ DC+DN+PR+SD+DC*DN | 9 | -97.208 | 213.7 | 4.37 | 0.011 |
| FTC ~ DC+DN+GS+PR+DC*DN | 9 | -97.209 | 213.7 | 4.37 | 0.011 |
| FTC ~ DC+DN+SD+DC*DN | 8 | -98.349 | 213.7 | 4.39 | 0.010 |
| FTC ~ DC+DN+GS+SD+DC*DN | 8 | -98.352 | 213.7 | 4.40 | 0.010 |
| FTC ~ GS | 4 | -102.722 | 213.7 | 4.41 | 0.010 |
| FTC ~ SD | 4 | -102.741 | 213.8 | 4.44 | 0.010 |
| FTC ~ DC+DN+PR+SD+DC*DN+DC*PR | 12 | -93.798 | 213.8 | 4.53 | 0.010 |
| FTC ~ DC+DN+GS+PR+DC*PR | 10 | -96.191 | 213.9 | 4.63 | 0.009 |
| FTC ~ DC+DN+PR | 7 | -99.603 | 214.0 | 4.67 | 0.009 |
| FTC ~ DC+DN+GS+PR+SD+DC*DN+DC*PR | 12 | -93.890 | 214.0 | 4.71 | 0.009 |
| FTC ~ DN+PR+DN*PR | 8 | -98.608 | 214.2 | 4.91 | 0.008 |
| FTC ~ DC+DN+PR+DC*PR+DN*PR | 11 | -95.173 | 214.2 | 4.92 | 0.008 |
| FTC ~ DC+DN+PR+DN*PR | 9 | -97.590 | 214.4 | 5.14 | 0.007 |
| FTC ~ DC+PR | 6 | -101.049 | 214.7 | 5.37 | 0.006 |
| FTC ~ DN+SB | 6 | -101.105 | 214.8 | 5.48 | 0.006 |
| FTC ~ DC+DN+SB | 7 | -100.008 | 214.8 | 5.48 | 0.006 |
| FTC ~ DC+DN+PR+SD+DC*PR | 11 | -95.484 | 214.9 | 5.54 | 0.006 |
| FTC ~ GS+PR | 6 | -101.262 | 215.1 | 5.79 | 0.005 |
| FTC ~ DC+DN+SD+SB+DC*DN | 9 | -97.920 | 215.1 | 5.80 | 0.005 |
| FTC ~ DC+DN+PR+SB+DC*DN | 10 | -96.776 | 215.1 | 5.80 | 0.005 |
| FTC ~ DC+PR+SD+DC*PR | 9 | -97.926 | 215.1 | 5.81 | 0.005 |
| FTC ~ DC+DN+PR+SD+DC*DN+DC*PR+DN*PR | 13 | -93.243 | 215.1 | 5.81 | 0.005 |
| FTC ~ SB | 5 | -102.363 | 215.1 | 5.83 | 0.005 |
| FTC ~ PR+SD | 6 | -101.290 | 215.2 | 5.85 | 0.005 |
| FTC ~ DC+DN+GS+SB+DC*DN | 9 | -97.956 | 215.2 | 5.87 | 0.005 |
| FTC ~ DC+DN+SD | 7 | -100.206 | 215.2 | 5.88 | 0.005 |
| FTC ~ DC+GS | 5 | -102.390 | 215.2 | 5.88 | 0.005 |
| FTC ~ DN+SD | 6 | -101.317 | 215.2 | 5.90 | 0.005 |
| FTC ~ DC+DN+GS+PR+DC*DN+DC*PR+DN*PR | 13 | -93.302 | 215.2 | 5.93 | 0.005 |
| FTC ~ DC+SD | 5 | -102.417 | 215.2 | 5.94 | 0.005 |
| FTC ~ DC+DN+PR+SB+DC*PR | 11 | -95.706 | 215.3 | 5.99 | 0.005 |
| FTC ~ DC+GS+PR+DC*PR | 9 | -98.028 | 215.3 | 6.01 | 0.005 |
| FTC ~ DC+DN+PR+SD+DC*DN+DN*PR | 11 | -95.741 | 215.4 | 6.06 | 0.005 |
| FTC ~ DC+DN+PR+SD+SB+DC*DN+DC*PR+ | 13 | -93.376 | 215.4 | 6.08 | 0.005 |
| FTC ~ DC+DN+GS+PR+SB+DC*DN+DC*PR | 13 | -93.385 | 215.4 | 6.10 | 0.004 |
| FTC ~ DC+DN+GS+PR+DC*DN+DN*PR | 11 | -95.783 | 215.5 | 6.14 | 0.004 |
| FTC ~ DN+GS+PR | 7 | -100.363 | 215.5 | 6.19 | 0.004 |
| FTC ~ DN+GS++SD | 6 | -101.481 | 215.5 | 6.23 | 0.004 |
| FTC ~ DN+PR+SD | 7 | -100.386 | 215.5 | 6.24 | 0.004 |
| FTC ~ DC+DN+GS+SD | 7 | -100.418 | 215.6 | 6.30 | 0.004 |
| FTC ~ PR+SB | 7 | -100.455 | 215.7 | 6.38 | 0.004 |
| FTC ~ GS+SD | 5 | -102.710 | 215.8 | 6.52 | 0.004 |
| FTC ~ DC+DN+GS+PR+SD+DC*PR | 11 | -95.984 | 215.9 | 6.54 | 0.004 |
| FTC ~ DC+DN+PR+SD+DC*DN | 10 | -97.195 | 216.0 | 6.64 | 0.003 |
| FTC ~ DC+DN+GS+SD+DC*DN | 9 | -98.348 | 216.0 | 6.65 | 0.003 |
| FTC ~ DC+DN+GS+PR+SD+DC*DN | 10 | -97.208 | 216.0 | 6.67 | 0.003 |
| FTC ~ DC+DN+PR+SB+DC*DN+DC*PR+DN*PR | 14 | -92.462 | 216.0 | 6.68 | 0.003 |
| FTC ~ DC+PR+SB+DC*PR | 10 | -97.217 | 216.0 | 6.68 | 0.003 |
| FTC ~ DC+DN+PR+SD | 8 | -99.528 | 216.1 | 6.75 | 0.003 |
| FTC ~ DC+DN+PR+SD+DC*PR+DN*PR | 12 | -94.929 | 216.1 | 6.79 | 0.003 |
| FTC ~ DC+DN+GS+PR | 8 | -99.601 | 216.2 | 6.90 | 0.003 |
| FTC ~ DC+SB | 6 | -101.815 | 216.2 | 6.90 | 0.003 |
| FTC ~ DC+DN+GS+PR+SD+DC*DN+DC*PR | 13 | -93.794 | 216.2 | 6.92 | 0.003 |
| FTC ~ DC+DN+PR+SD+DN*PR | 10 | -97.337 | 216.2 | 6.92 | 0.003 |
| FTC ~ DC+PR+SB | 8 | -99.661 | 216.3 | 7.02 | 0.003 |
| FTC ~ DN+GS+PR+DN*PR | 9 | -98.579 | 216.4 | 7.12 | 0.003 |
| FTC ~ DN+PR+SD+DN*PR | 9 | -98.588 | 216.4 | 7.13 | 0.003 |
| FTC ~ DC+DN+GS+PR+DC*PR+DN*PR | 12 | -95.163 | 216.6 | 7.26 | 0.002 |
| FTC ~ DC+DN+GS+PR+DN*PR | 10 | -97.588 | 216.7 | 7.43 | 0.002 |
| FTC ~ DC+PR+SD | 7 | -100.999 | 216.8 | 7.47 | 0.002 |
| FTC ~ DC+GS+PR | 7 | -101.031 | 216.8 | 7.53 | 0.002 |
| FTC ~ DC+DN+PR+SB | 9 | -98.806 | 216.9 | 7.57 | 0.002 |
| FTC ~ DN+GS+SB | 7 | -101.055 | 216.9 | 7.58 | 0.002 |
| FTC ~ DN+SD+SB | 7 | -101.058 | 216.9 | 7.58 | 0.002 |
| FTC ~ DN+PR+SB | 8 | -99.956 | 216.9 | 7.61 | 0.002 |
| FTC ~ DC+DN+PR+SB+DC*DN+DN*PR | 12 | -95.348 | 216.9 | 7.63 | 0.002 |
| FTC ~ DC+DN+GS+SB | 8 | -99.998 | 217.0 | 7.69 | 0.002 |
| FTC ~ DC+DN+SD+SB | 8 | -100.004 | 217.0 | 7.71 | 0.002 |
| FTC ~ GS+SB | 6 | -102.284 | 217.1 | 7.84 | 0.002 |
| FTC ~ SD+SB | 6 | -102.296 | 217.2 | 7.86 | 0.002 |
| FTC ~ DC+DN+GS+PR+SD+DC*PR | 12 | -95.470 | 217.2 | 7.87 | 0.002 |
| FTC ~ GS+PR+SD | 7 | -101.251 | 217.3 | 7.97 | 0.002 |
| FTC ~ DC+GS+PR+SD+DC*PR | 10 | -97.862 | 217.3 | 7.97 | 0.002 |
| FTC ~ DC+GS+SD | 6 | -102.388 | 217.4 | 8.05 | 0.002 |
| FTC ~ DC+DN+SD+SB+DC*DN | 10 | -97.919 | 217.4 | 8.09 | 0.002 |
| FTC ~ DC+DN+GS+SD+SB+DC*DN | 10 | -97.919 | 217.4 | 8.09 | 0.002 |
| FTC ~ DN+GS+SD | 7 | -101.312 | 217.4 | 8.09 | 0.002 |
| FTC ~ DC+DN+PR+SD+SB+DC*PR | 12 | -95.578 | 217.4 | 8.09 | 0.002 |
| FTC ~ DC+DN+GS+SD | 8 | -100.206 | 217.4 | 8.11 | 0.002 |
| FTC ~ DC+DN+PR+SD+SB+DC*DN | 11 | -96.775 | 217.4 | 8.13 | 0.002 |
| FTC ~ DC+DN+GS+PR+SB+DC*DN | 11 | -96.775 | 217.4 | 8.13 | 0.002 |
| FTC ~ DC+DN+PR+SD+DC*DN+DC*PR+DN*PR | 14 | -93.210 | 217.5 | 8.18 | 0.002 |
| FTC ~ DC+DN+GS+PR+SD+DC*DN+DC*PR+DN*PR | 14 | -93.232 | 217.5 | 8.22 | 0.002 |
| FTC ~ DN+PR+SD | 8 | -100.302 | 217.6 | 8.30 | 0.001 |
| FTC ~ DC+DN+GS+PR+SB+DC*PR | 12 | -95.685 | 217.6 | 8.31 | 0.001 |

**Table S4.** Flight-initiation distances of European Rabbits (*Oryctolagus cuniculus*)*.*

| **Location** | **Status of rabbit population** | **Approach stimulus, during day and/ or night** | **Mean ± SE FID (m)** | **Source** |
| --- | --- | --- | --- | --- |
| Victoria, Australia* | Introduced | Human, during day and night | 30.37 ± 1.83 | This study** |
| Bristol, England, UK* | Native | Human, during day | 31.50 ± 0.44 | Huang et al. 2025; Huang 2024 |
| Bristol, England, UK | Native | Red fox *Vulpes vulpes* (taxidermied), during day | 30.00 ± 1.37 | Huang et al. 2025; Huang 2024 |
| Bristol, England, UK | Native | Buzzard, *Buteo buteo* (live), during day | 20.50 ± 1.64 | Huang et al. 2025; Huang 2024 |
| Bristol, England, UK | Native | Pine Marten *Martes martes* (taxidermied), during day | 21.50 ± 1.31 | Huang et al. 2025; Huang 2024 |
| Bristol, England, UK | Native | European rabbit, *O. cuniculus* (taxidermied), during day | 16.50 ± 1.50 | Huang et al. 2025 |
| Spyknoll (30 km south of Aberdeen), Scotland, UK | Native | Human, during day | Open habitat:  62 ± 1.24 m (adults); Scrub (closed):  46 ± 1.73 m (adults) | Boyce 1984 |
| Spyknoll | Native | Dog (live), during day | *c.* 26 ± 1.67 m, Adult  *c.* 16 ± 1.45 m, Young | Boyce 1984 |
| Spyknoll | Native | Ferret (live), during day | *c.* 5 ± 2.55 m, Adult  *c.* 4 ± 2.33 m, Young | Boyce 1984 |

* The rabbits introduced into Australia (near Geelong in Victoria) that became invasive were sourced from around Baltonsborough, Somerset, southwest England (Rolls 1969; Alves et al. 2022), 50 km south of Bristol (UK).

** Also see Rendall et al. (2024).

**References**

Alves JM, Carneiro M, Day JP, Welch JJ, Duckworth JA, Cox TE, Letnic M, Strive T, Ferrand N, Jiggins FM (2022). A single introduction of wild rabbits triggered the biological invasion of Australia. *Proceedings of the National Academy of Sciences of the United States of America* 119(35), e2122734119.

Boyce JL (1984). The defense against predation of the European rabbit *Orychtolagus cuniculus*. Unpubl. PhD thesis, University of Aberdeen, Scotland, U.K.

Huang Y (2024). Antipredator Defences, with Particular Relevance to European Rabbits. Unpublished PhD dissertation, University of Bristol, UK: <https://research-information.bris.ac.uk/ws/portalfiles/portal/445823288/Final_Copy_2024_10_01_Huang_Y_MScR_Redacted.pdf>

Huang Y, Sparke RE and Caro T (2025). The adaptive significance of tail‐flagging: a test in European Rabbits (*Oryctolagus cuniculus*). *Ecology and Evolution.* *15*(6):p.e71632

Rendall AR, Plotz RD, Yokochi K, Krauss J, Pengelly A, Di Stefano SA, Swindell S, Ranawana K, Vidanapathirana DR and Weston, MA (2024). Lifting the Veil of Darkness: Thermal Technology Facilitates Collection of Flight‐Initiation Distances by Night. *Ecology and Evolution.* 14(11):p.e70450.

Rolls EC (1969). They all ran wild: the story of pests on the land in Australia. Angus and Robertson. London.
